# Vitamin D_3_ replacement enhances antigen-specific immunity in older adults

**DOI:** 10.1093/immadv/ltaa008

**Published:** 2020-11-25

**Authors:** Emma S Chambers, Milica Vukmanovic-Stejic, Carolin T Turner, Barbara B Shih, Hugh Trahair, Gabriele Pollara, Evdokia Tsaliki, Malcolm Rustin, Tom C Freeman, Neil A Mabbott, Mahdad Noursadeghi, Adrian R Martineau, Arne N Akbar

**Affiliations:** Centre for Immunobiology, Blizard Institute, Queen Mary University of London, London, UK; Division of Infection and Immunity, University College London, London, UK; Division of Infection and Immunity, University College London, London, UK; Division of Infection and Immunity, University College London, London, UK; The Roslin Institute and Royal (Dick) School of Veterinary Studies, University of Edinburgh, Easter Bush, Midlothian, UK; Division of Medicine, University College London, London, UK; Division of Infection and Immunity, University College London, London, UK; Division of Infection and Immunity, University College London, London, UK; Department of Dermatology, Royal Free Hospital, London, UK; The Roslin Institute and Royal (Dick) School of Veterinary Studies, University of Edinburgh, Easter Bush, Midlothian, UK; The Roslin Institute and Royal (Dick) School of Veterinary Studies, University of Edinburgh, Easter Bush, Midlothian, UK; Division of Infection and Immunity, University College London, London, UK; Centre for Immunobiology, Blizard Institute, Queen Mary University of London, London, UK; Division of Medicine, University College London, London, UK

**Keywords:** vitamin D, varicella zoster virus, skin, ageing

## Abstract

**Introduction:**

Ageing is associated with increased number of infections, decreased vaccine efficacy and increased systemic inflammation termed inflammageing. These changes are reflected by reduced recall responses to varicella zoster virus (VZV) challenge in the skin of older adults. Vitamin D deficiency is more common in the old and has been associated with frailty and increased inflammation. In addition, vitamin D increases immunoregulatory mechanisms and therefore has the potential to inhibit inflammageing.

**Objectives:**

We investigated the use of vitamin D_3_ replacement to enhance cutaneous antigen-specific immunity in older adults (≥65 years).

**Methods:**

Vitamin D insufficient older adults (*n* = 18) were administered 6400IU of vitamin D_3_/day orally for 14 weeks. Antigen-specific immunity to VZV was assessed by clinical score assessment of the injection site and transcriptional analysis of skin biopsies collected from challenged injection sites pre- and post-vitamin D_3_ replacement.

**Results:**

We showed that older adults had reduced VZV-specific cutaneous immune response and increased non-specific inflammation as compared to young. Increased non-specific inflammation observed in the skin of older adults negatively correlated with vitamin D sufficiency. We showed that vitamin D_3_ supplementation significantly increased the response to cutaneous VZV antigen challenge in older adults. This enhancement was associated with a reduction in inflammatory monocyte infiltration with a concomitant enhancement of T cell recruitment to the site of antigen challenge in the skin.

**Conclusion:**

Vitamin D_3_ replacement can boost antigen-specific immunity in older adults with sub-optimal vitamin D status.

## Graphical Abstract

**Graphical Abstract:**
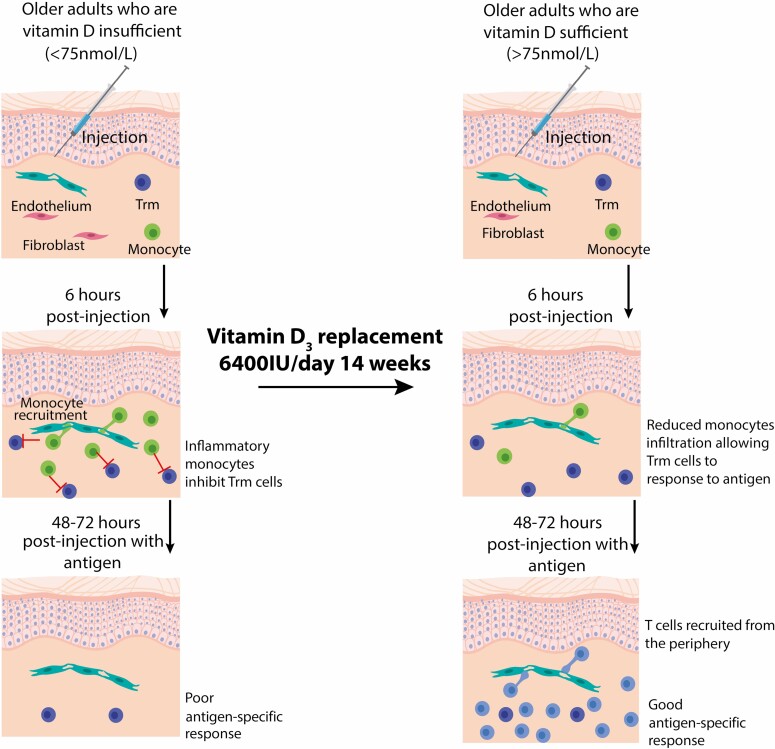

## Introduction

Immunity decreases during ageing as demonstrated by the increased susceptibility to bacterial and viral infections, re-activation of latent infections such as varicella zoster virus (VZV), decreased vaccine efficacy, and increased incidence of cancer [1–3]. In addition, there is an increase in low-grade systemic inflammation in older humans termed inflammageing. This is characterised by high serum levels of the inflammatory cytokines IL-6, IL-1β, TNFα, and C reactive protein (CRP) [4], and is a strong predictor for frailty and mortality [5, 6]. Inflammageing is also believed to contribute to reduced antigen-specific immunity that is observed with older age (≥65 years) [7, 8].

Antigen-specific cutaneous recall responses are reduced in healthy old as compared to young individuals [8–11]. We have shown that intradermal injections of air, saline, or antigen into the skin of older adults are associated with induction of an early non-specific inflammation which directly contributes to reduced cutaneous immunity [12]. We proposed that this non-specific inflammation is driven by senescent fibroblasts recruiting inflammatory monocytes that secrete PGE_2_ and directly inhibit antigen-specific immunity [12]. Blockade of inflammation using the anti-inflammatory drug Losmapimod (a specific p38 MAP kinase inhibitor) can restore antigen-specific immunity in older adults via inhibition of the non-specific inflammation in the skin [8, 12].

Vitamin D has key immunomodulatory properties including increasing the abundance of regulatory T cells (Tregs) [13–15], reducing inflammatory cytokine production by T cells and monocytes [16, 17] as well as increasing antimicrobial peptide production [18]. Vitamin D insufficiency, as determined by serum 25-hydroxyvitamin D (25[OH]D) levels <75 nmol/l, is more common in the older adult (>65 years) population, particularly in those who are frail and who have elevated inflammatory markers [19–21]. Therefore, vitamin D insufficiency may exacerbate inflammageing and non-specific inflammation observed in older adults.

As vitamin D insufficiency is associated with ageing and inflammation, we initiated a clinical study using vitamin D replacement in older adults with sub-optimal vitamin D status to assess if vitamin D_3_ replacement improves secondary cutaneous immunity. Older adults with vitamin D insufficiency (25(OH)D <75 nmol/l), were orally administered 6400 IU of vitamin D_3_ per day for 14 weeks. Antigen-specific immunity was assessed by measuring the clinical response to VZV challenge and by transcriptional analysis of skin biopsies collected pre- and post-vitamin D_3_ replacement. We show that vitamin D_3_ replacement can significantly improve VZV-specific cutaneous immunity in older adults. Vitamin D therefore has the potential to be used as a cheap, safe, and effective therapy to enhance antigen-specific immunity in the skin of elderly humans.

## Materials and methods

### Study approval

This study was approved by the NHS Queen Square Research Ethics Committee (reference 17/SC/0196) and by the UCL Research Ethics Committee. All participants provided written informed consent, and study procedures were performed in accordance with the principles of the declaration of Helsinki. We were advised by the UK’s Medicines and Healthcare products Regulatory Agency (MHRA) that the study was not classified as a Clinical Trial of an Investigational Medicinal Product (IMP) as defined by the EU Directive 2001/20/EC. As this experimental medicine study was designed to test a hypothesis in humans *in vivo* and not to determine the therapeutic outcome or efficacy of vitamin D_3_ for patient benefit.

### Study participants

**Figure 1.**
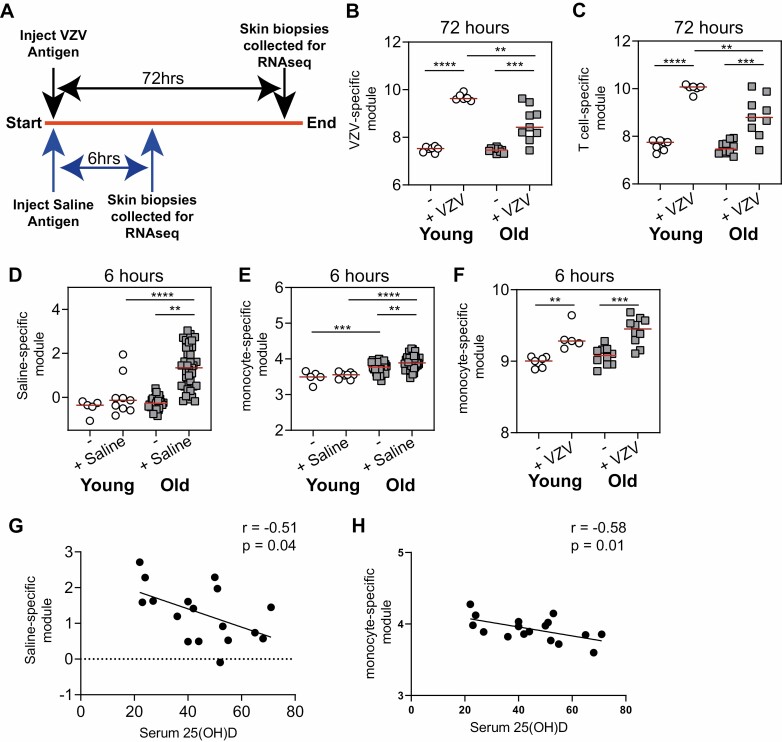
Decreased cutaneous immunity with age correlates with vitamin D insufficiency. (A) study schematic, young (white) and old (grey) individuals were injected with either antigen or saline and biopsies were collected at specified time points and RNAseq or microarray analysis was performed. Samples were compared to normal [unmanipulated; - (young *n* = 5 and old *n* = 32)] skin. (B) Antigen-specific gene module was generated and (C) T cell-specific gene module in VZV-injected skin (72 hours post-injection; young *n* = 6 and young *n* = 9). (D) saline-specific gene module and (E) monocyte-specific gene module in saline injected skin (6 hours post-injection; young *n* = 9 and old *n* = 37). (F) Monocyte-specific gene module in VZV injected skin (6 hours post-injection; young *n* = 6 and young *n* = 9). (G) Saline-specific module and (H) monocyte-specific module in saline-injected skin from old donors was correlated with serum 25(OH)D concentrations (nmol/l). B–F were analysed with an unpaired *t* test and G and H were analysed by a Pearson correlation test. **P < 0.01; ***P < 0.001; ****P < 0.0001.

For the study involving young (<40 years) and old (≥65 years) adults (Fig. 1), we recruited healthy individuals of white European ethnicity. We excluded individuals with co-morbidities that are associated with significant internal organ or immune dysfunction including heart failure, severe chronic obstructive pulmonary disease, diabetes mellitus and rheumatoid arthritis, and individuals receiving immunosuppressive treatment (e.g. oral glucocorticoids, methotrexate, azathioprine, and cyclosporin) for autoimmune or chronic inflammatory diseases.

**Figure 2.**
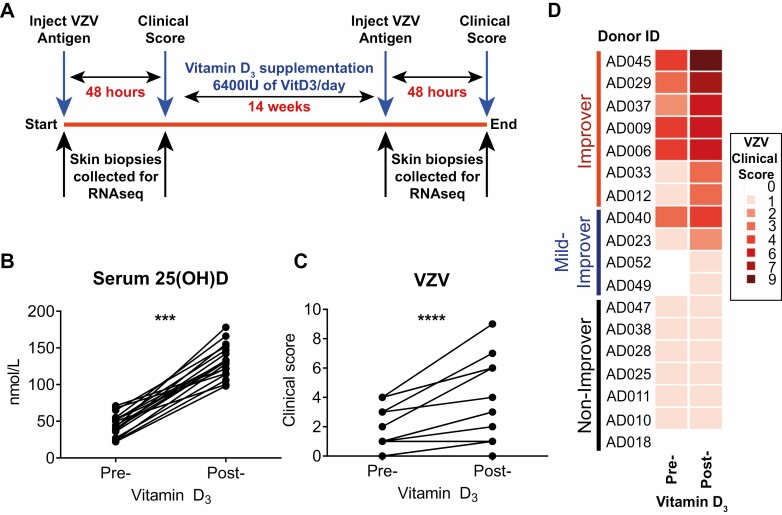
Vitamin D_3_ supplementation significantly improves VZV-specific cutaneous immunity. (A) Clinical study schematic. (B) Serum 25(OH)D concentrations and C and D, VZV clinical scores in older adults pre- and post-supplementation (*n* = 18). B and C were analysed with a paired *t* test. ***P < 0.001; ****P < 0.0001.

**Figure 3.**
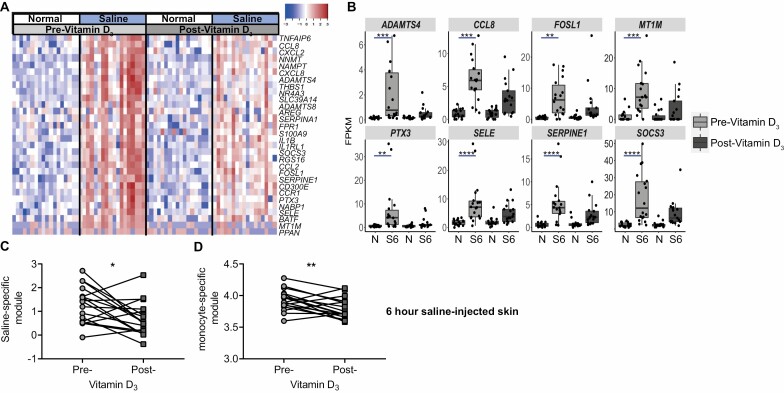
Vitamin D_3_ supplementation is associated with reduced inflammatory monocyte recruitment in response to saline. (A) RNAseq analysis of 3 mm biopsies collected from normal and saline-injected skin (6 hours post-injection) pre- and post-vitamin D_3_ supplementation. The top 30 genes upregulated in saline-injected skin as compared to normal skin before pre-Vitamin D_3_ and (B) dot plots of top eight upregulated saline-associated genes pre-vitamin D_3_. (C) Saline-specific module and (D) monocyte-specific module in saline-injected skin pre- and post-vitamin D_3_ supplementation (*n* = 17). B, analysed by C and D, analysed with a Wilcoxon-matched paired test. *P < 0.05; **P < 0.01; ***P < 0.001; ****P < 0.0001.

**Figure 4.**
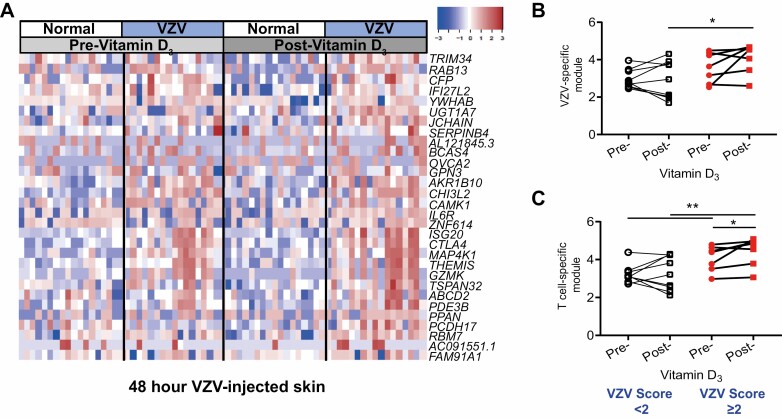
Vitamin D_3_ supplementation increases the accumulation of T cells at the site of VZV challenge. (A) RNAseq analysis of 3 mm biopsies collected from normal and VZV-injected skin (48 hours post-injection) pre- and post-vitamin D. The top 30 genes upregulated in VZV injected skin as compared to normal skin before post-Vitamin D_3_ (*n* = 16). (B) VZV-specific module and (C) T cell specific module in VZV-injected skin pre- and post-vitamin D_3_ supplementation separated based upon improvement in VZV score change of <2 (white; *n* = 9) and change ≥2 (red; *n* = 7). Paired data were analysed using a Wilcoxon-matched paired test and unpaired data with Mann–Whitney test. *P < 0.05; **P < 0.01.

For the study involving vitamin D_3_ (Figs. 2–4), healthy older adults were recruited to take part through local GP surgeries. When individuals expressed an interest in the study, they were screened and recruited according to the inclusion and exclusion criteria (Supplementary Table 1). We recruited 18 healthy older individuals, VZV skin test and saline injection were performed and biopsies were collected at 6 and 48 hours. Subsequently, individuals were given 6400 IU of vitamin D_3_ per day for 14 weeks orally. After vitamin D_3_ supplementation, the participants repeated the same VZV skin test and skin biopsies were collected as before (Fig. 3A). Serum CRP levels were measured using a Roche cobas high-sensitive immunoturbidimetric assay, and 25(OH)D concentrations were measured with a Roche cobas electrochemiluminescence immunoassay.

### Skin tests

VZV antigen (BIKEN, The Research Foundation for Microbial Diseases of Osaka University, Japan) or 0.9% saline solution were injected intradermally into sun unexposed skin of the medial proximal volar forearm as per the manufacturer’s instructions. Induration, palpability, and the change in erythema from baseline were measured and scored on Day 2 or 3 as validated and described previously [11]. A clinical score (range 0–10) based on the summation of these parameters was then calculated.

### RNAseq analysis of skin biopsies

Three separate 3 mm punch biopsies were collected from each volunteer: one from normal (un-injected) skin, one from the saline injection site at 6 hours post-injection and one from the VZV injection site at 48 or 72 hours post-injection. Biopsies were immediately stabilised in RNAlater for cryostorage. Total RNA was extracted from bulk tissue homogenates using RNeasy Mini Kit (Qiagen) as previously described [8]. Library preparation for RNAseq was performed using the Kappa Hyperprep kit (Roche Diagnostics) and sequencing was performed by the Pathogens Genomic Unit (UCL) on the Illumina Nextseq 500 (Illumina) using the NextSeq 500/550 High Output 75 cycle kit (Illumina) according to the manufacturers’ instructions, resulting in a median of 22.7 million (range 1.4–38.6 million; IQR 20.8–24.4 million) 41 bp paired-end reads per sample.

### Module analysis

RNAseq data were mapped to the reference transcriptome (Ensembl Human GRCh38 release 99) using Kallisto [22]. The transcript-level output counts and transcripts per million (TPM) values were summed on gene level and annotated with Ensembl gene ID, gene name, and gene biotype using the R/Bioconductor packages tximport and BioMart [23, 24]. Downstream analyses were restricted to gene biotypes with selected BioMart annotations (protein coding, IG_C_gene, IG_D_gene, IG_J_gene, IG_V_gene, TR_C_gene, TR_D_gene, TR_J_gene, TR_V_gene), resulting in 23,402 Ensembl gene IDs.

Heatmap and individual gene analysis: Reads were aligned to Genome Reference Consortium Human Build 38 (GRCh38) using Hisat2 [25]. Samtools was used to select for reads with paired mates. Transcript assembly was carried out using StringTie [26], with gene-level Fragments per Kilobase of transcript per Million mapped read (FPKM) generated using Ballgown [27]. Statistical comparisons were made on gene count estimates generated by StringTie. Genes with low expression or short transcript lengths (<200 nucleotides for the longest transcript) were removed. The count matrix was normalised using the TMM method in edgeR (version 3.22.5) [28], followed by contrast fit with voom in limma (version 3.36.5) [29], treating the subject ID as a blocking variable. Genes with an adjusted *P*-value of less than 0.05 and expression change of greater than 2-fold up or down, were considered to be statistically significant.

### Microarray data

Data from previous microarray experiments were utilised in this study [8]. Following robust multi-array average normalisation with the R/Bioconductor package affy [30], only unique gene name annotations were retained, selecting the probe ID with highest average expression across all samples.

### Transcriptional modules

The gene expression modules for T cells and monocytes have been described [31, 32] and validated previously [33]. The VZV-specific model was generated by the mean expression of genes in a transcriptional module comprising differential gene expression in biopsies from the site of VZV injection in young adults as compared to normal (unmanipulated) skin. The saline-specific module was represented by the mean expression of genes in a transcriptional module comprising differentially gene expression in biopsies from the site of saline injection in old individuals as compared to normal skin. In each case, differentially expressed genes with false discovery rate <0.05 and log2 fold difference ≥1were identified using DeSeq2 and SARTools [34] for RNAseq data, and Mann–Whitney tests in MultiExperiment Viewer v4.9 (http://www.tm4.org/mev.html) for microarray data, based on false discovery rate <0.05 and log2 fold difference ≥1. Gene module scores were subsequently calculated as mean expression of the constituent gene names in each module. For RNAseq data, log2-transformed TPM values were used, following the addition of a pseudocount of 0.001 to enable log2 transformation. Where duplicate gene names were present in the RNAseq data, the highest log2 TPM value was used for each sample.

Reactome pathway enrichment among module genes was analysed with the XGR R package [35]. For visualisation purposes, 20 pathway groups were identified by hierarchical clustering of Jaccard indices to quantify similarity between the gene composition of each pathway. For each group, the pathway with the largest total number of genes was then selected to provide a representative annotation.

### Serum cytokine measurements

Cytokine concentration in serum was assessed by cytometric bead array (BD Biosciences) according to the manufacturer’s protocol. Samples were analysed using a BD Verse flow cytometer (BD Biosciences). The lower limit of detection for each analyte was 1.5 pg/ml.

### Statistics

Statistical analysis was performed using GraphPad Prism version 8.00 (GraphPad Software, San Diego, CA, USA). Data were assessed for normality and the subsequent appropriate statistical test was performed as indicated in the legend of each figure.

## Results

### Low serum 25-hydroxyvitamin D concentrations correlate with inflammatory response to saline

We have shown previously that older adults exhibit an early non-specific inflammatory response to intradermal injection which is associated with a reduced delayed-type hypersensitivity responses to the VZV skin test [9]. We sought to extend these findings by performing modular bioinformatic analysis, as validated previously [33]. We intradermally challenged healthy young (<40 years) and old (≥65 years) individuals with VZV antigen (in individuals who had pre-existing VZV immunity); for donor characteristics, see Table 1). The site of challenge in the skin was biopsied 72 hours later and RNAseq or microarray analysis was performed and compared to normal, unmanipulated, skin (Fig. 1A). In line with our previous studies [8, 12], 6-hour saline injection was used as a control for non-specific (needle injury) responses.

**Table 1.** Donor characteristics of young and old donors

Characteristics	Normal		VZV		Saline	
	Young	Old	Young	Old	Young	Old
Age	19.0 (18–23)	69.0 (65–82)	25.5 (20–27)	74.0 (66–83)	19.0 (18–23)	69.0 (65–82)
Gender	3 Male 2 Female	14 Male 18 Female	5 Male 1 Female	2 Male 7 Female	5 Male 4 Female	17 Male 20 Female
Serum 25(OH)D (nmol/l)	49 (29–88)	50.5 (25–103)	No data	No data	54.0 (35–88)^1^	52.5 (26–108)
Number of donors	5	32	6	9	9	37

Data shown as median ± 10-90 Percentile.

Normal = unmanipulated biopsied skin; VZV, varicella zoster virus.

^1^Three donors had no serum 25(OH)D measurements.

We derived transcriptional modules (signatures) to quantify the VZV-specific cutaneous immune response (Supplementary Fig. 1A and Supplementary Table 2). As expected, the expression of genes within the VZV-specific module was increased in young and old adults after skin challenge [8]. However, the magnitude of the secondary response to VZV antigen was significantly lower in older individuals compared to the young individuals (Fig. 1B). In a previous study, we have observed that there was a significant accumulation of T cells at the site of VZV antigen challenge in young subjects which was greatly reduced in older adults [36]. To identify if T cells are as important for a VZV response, expression of a previously generated T cell-specific gene module was used [33]. We observed that following injection with VZV, there was a significant increase in expression of the T cell-specific module (Fig. 1C). Since the magnitude of expression of the T cell-specific module correlates directly with the number of T cells present [33], this suggested that there was an increase in T cell numbers in antigen-injected skin as compared to normal skin. Indeed, our analysis showed that the expression level of the genes in the T cell-specific module correlated directly with the magnitude of the VZV clinical score (Supplementary Fig. 2).

We previously showed that a monocyte-driven inflammatory response to injection is responsible for the impaired T cell response to VZV in the skin of older individuals [8, 12]. We therefore created a gene module associated with non-specific saline injection based upon gene expression in 6-hour saline-injected old skin. This saline-specific module was enriched in genes and pathways associated with the innate immune system and interleukin signalling (Supplementary Fig. 1B and Supplementary Table 3). We confirmed that there was a significant induction of an inflammatory response in saline-injected old skin that was not observed in the young (Fig. 1D). Consistent with our previous observation, we found that enrichment of a monocyte-specific module was significantly greater in saline injection sites of older compared to younger individuals (Fig. 1E). Expression of the monocyte-specific module was also increased in the skin of older adults 6 hours after injection with VZV antigen (Fig. 1F), confirming the non-specific recruitment of monocytes to the tissue damage caused by needle injection rather than specific to saline [12].

Next, in order to evaluate the potential role of vitamin D in inflammageing, we sought to understand if vitamin D insufficiency was associated with the exaggerated non-specific monocytic inflammatory response to saline injection we found in older individuals. In keeping with this, we found that there was a significant negative correlation between serum 25(OH)D concentrations and both the expression of the saline-induced transcriptional module and the monocyte module in older adults (Fig. 1G and H).

Therefore, these data suggest that vitamin D insufficiency is associated with increased non-specific inflammation in the skin of older adults.

### Vitamin D_3_ supplementation significantly improved cutaneous secondary immune response in older adults.

We hypothesised that if vitamin D insufficiency may be causally related to inflammageing, and in turn mechanistically linked to attenuation of antigen specific recall responses, then vitamin D supplementation may rescue age-related diminution of immune responses. We tested this hypothesis by evaluating immune responses before and after of vitamin D replacement (6400 IU of vitamin D_3_ per day orally for 14 weeks) among older adults (median age 69 years; 6 males and 12 females), with low concentrations of serum 25(OH)D [median 43 nmol (22.9–68.3 nmol/l)] (Fig. 2A). We utilised 6400 IU/day in order to maximise our chances of elevating circulating 25(OH)D levels into high physiological range, without risking toxicity by exceeding the tolerable upper intake level (UL) of 10,000 IU/day [37]. All older adults had a significant increase in their serum 25(OH)D concentrations after vitamin D replacement (Fig. 2B) confirming compliance with the vitamin D supplementation regime. We observed a significant increase in VZV clinical scores after vitamin D supplementation (Fig. 2C and D) using an ordinal scale clinical score [11]. The increase in VZV clinical score was not due to repeated exposure of antigen, as we have shown previously that repeated exposure to VZV antigen over the same time frame as used in this study, does not increase VZV clinical score [8].

We further stratified the participants into three groups based on the magnitude of their clinical response following vitamin D_3_ supplementation: non-improvers, who did not have an improvement in clinical score; mild-improvers, clinical score improved by 1; improvers, those who had an improvement in their clinical score of ≥2 (Fig. 2D). Analysis of the characteristics of each of these groups revealed that there were no significant differences in their ages, serum 25(OH)D, or CRP concentrations at baseline (Table 2) or after vitamin D supplementation. There was, however, an increased proportion of females in the improvers when compared to the other two groups (Table 2).

**Table 2.** Donor characteristics

Donor characteristics	Non-improver (NI)	Mild-improver (MI)	Improver (I)	Significant?
Age	70 (65–82)	73 (68–81)	69 (65–69)	ns
Gender	3 Male; 4 Female	2 Male; 2 Female	1 Male; 6 Female	
VZV clinical score at baseline	1	0.5	3	***NI vs. I
CRP at baseline (mg/l)	0.8 (0.3–24.3)	0.7 (0.3–24.3)	0.8 (0.4–2.6)	ns
Serum 25(OH)D at baseline (nmol/l)	40.0 (23–68)	53.0 (37–65)	42.0 (22–71))	ns
Serum 25(OH)D after Vitamin D_3_ supplementation (nmol/l)	89.0 (47–102)	103.5 (87–118)	78.0 (50.0–136.0)	ns
Change in clinical score	0	1	2	*** NI vs. I
Number of donors	7	4	7	

Non-improvers VZV clinical score change of 0, mild improvers VZV clinical score change of 1 and improvers VZV clinical score change of >1 after vitamin D3 supplementation. Data shown as median ± 10-90 Percentile. Data analysed by Kruskal–Wallis test.

ns, non-significant; VZV, varicella zoster virus.

***P < 0.001.

These data suggest that vitamin D replacement can significantly enhance antigen-specific immunity during ageing.

### Vitamin D_3_ supplementation decreased non-specific monocyte-driven inflammation

Following 14 weeks of vitamin D_3_ replacement, there was no significant impact on circulating inflammatory cytokine or CRP concentrations (Table 3). This suggested that the beneficial anti-inflammatory effect of vitamin D_3_ is specific to the site of antigen challenge in the skin. Next, we evaluated the effect of vitamin D_3_ supplementation on the non-specific inflammatory response to saline injection. Three millimetre skin biopsies were collected from normal and saline-injected skin (6 hours post-injection) pre- and post-vitamin D_3_ replacement. As observed previously [8, 12], there was a large proinflammatory response to saline injection in older adults which was characterised by increased expression of monocyte chemoattractants and cytokines such as *CCL2*, *CCL8*, and *IL1B*. The expression of these inflammatory genes was reduced after vitamin D_3_ supplementation (Fig. 3A). Focusing on the eight most upregulated genes in response to saline prior to vitamin D_3_ replacement, we observed that, after supplementation, these genes were no longer statistically significantly upregulated as compared to normal skin (Fig. 3B). Consistent with these findings, we found that expression of both the saline-induced and monocyte transcriptional modules were significantly decreased after vitamin D_3_ supplementation (Fig. 3C and D), suggesting that vitamin D_3_ supplementation can reduce the non-specific inflammation and the associated inflammatory monocyte recruitment which was associated with needle challenge in older adults.

**Table 3.** Serum inflammatory cytokines pre- and post-vitamin D3 supplementation

Cytokines	Pre-vitamin D_3_	Post-vitamin D_3_	*P* value
CCL2	12.1 pg/ml (8.47–15.7)	11.6 pg/ml (8.67–14.6)	0.99
IL-1β	0.17 pg/ml (0.00–0.40)	0.15 pg/ml (0.00–0.30)	0.84
IL-6	0.60 pg/ml (0.28–0.91)	0.54 pg/ml (0.22–0.85)	0.58
IL-8	6.58 pg/ml (4.36–8.82)	16.0 pg/ml (4.01–27.9)	0.14
IFNα	9.68 pg/ml (0.94–18.4)	8.97 pg/ml (0.00–18.4)	0.48
TNFα	0.41 pg/ml (0.01–0.80)	0.60 pg/ml (0.00–1.25)	0.67
CRP	2.42 mg/l (0.00–5.16)	1.98 mg/l (0.87–3.10)	0.73

Serum samples were collected pre- and post-vitamin D3 supplementation (*n* = 18). Cytokine concentrations were assessed by cytometric bead array. Data shown as mean ± 95% CI. Data were analysed by paired *t* test.

### Vitamin D supplementation enhances T cell accumulation in the skin after antigen challenge

We have previously shown that inflammatory monocytes recruited to the skin of older adults in response to needle challenge blocks antigen-specific T cell responses and that inhibiting monocyte infiltration can improve cutaneous immunity [12]. We wanted to investigate whether vitamin D_3_ supplementation could also reverse inflammatory monocytes recruitment and thus the attenuated T cell responses to VZV antigen in older adult skin. Specifically, we wanted to determine whether the decrease in monocyte infiltration following vitamin D_3_ supplementation leads to an enhancement of T cell accumulation at the site of antigen challenge. To assess this, gene expression in VZV-injected skin (48 hours after injection) was compared by RNAseq analysis pre- and post-vitamin D_3_ supplementation, and no significant differential overall gene expression was observed (Fig. 4A). We reasoned that the heterogeneity of the effect of vitamin D_3_ supplementation meant that our sample size was underpowered to detect statistically consistent differences in the whole group, particularly in view of the multiple testing penalty for gene-wide analysis. Therefore, we focused our analysis on VZV-induced and T cell transcriptional modules after stratifying participants by the vitamin D_3_ associated improvement in their VZV clinical score, into those who were non-improvers or mild improvers (clinical score change ≤1) as compared to improvers (clinical score change >1). We found that improvers had a significant increase in the expression of the VZV-specific module after vitamin D_3_ supplementation as compared to those who were mild/ non-improvers (Fig. 4B). In addition, the T cell-specific module was significantly increased in VZV-injected skin in the improvers but not in the mild/non-improvers (Fig. 4C). Interestingly, individuals whose VZV clinical score increased by ≥2 had a higher expression of the T cell module in response to VZV prior to vitamin D_3_ supplementation.

Collectively, our data are consistent with a mechanistic model in which vitamin D status may enhance antigen-specific immunity by reducing non-specific monocyte-driven inflammation and enhancing T cell-mediated recall responses.

## Discussion

In this study, we confirmed that antigen-specific cutaneous immune responses were reduced in the skin of older adults (≥65 years) when compared with young (<40 years). In agreement with our previous work, the reduced secondary cutaneous response was associated with an increased monocyte-derived non-specific inflammatory response to needle challenge in the older adults. As vitamin D has a role in controlling inflammation, we investigated whether vitamin D insufficiency correlated with the increased inflammatory response that occurs in the skin after needle challenge. There was increased non-specific inflammation in response to injection (determined by increased expression of genes in the saline response module) in individuals that were most vitamin D deficient. Furthermore, we demonstrated that vitamin D_3_ supplementation in older adults (6400 IU vitamin D_3_ per day for 14 weeks) significantly improved cutaneous secondary immune responses to VZV antigen. Our transcriptional analyses suggested that this increase in cutaneous immunity was associated with decreased early monocyte-driven inflammation and subsequent increased recruitment of T cells to the site of antigen challenge.

In this article, we confirm using bioinformatic modular analysis, our earlier observation that an early (6 hours) monocyte-driven non-specific inflammatory response is observed in older adults but not in the young [8, 12]. This non-specific inflammatory response is associated with worse antigen-specific cutaneous immunity, as characterised by reduced T cells present in VZV injected skin. Vitamin D_3_ replacement significantly reduced monocyte gene signatures in saline injected skin and increased T cell signatures in those individuals who had an improvement in their clinical score. These data propose that vitamin D_3_ supplementation inhibits monocyte recruitment to injected skin of older people and therefore limits monocyte-driven suppression of T resident memory (T_RM_) cells at the site of antigen challenge. It is interesting to note that the T cell signature only increases in VZV injected skin of individuals that had an improvement in their clinical score even though the non-specific inflammatory response is reduced in the majority of participants after vitamin D_3_ replacement. One reason for this might be that the T cell response is only increased in those individuals who have a more measurable T cell response to antigen prior to vitamin D_3_ supplementation.

Older adults have increased risk of mortality from primary infections such as influenza and the SARS-CoV-2 coronavirus, and have an increased risk of reactivation of persistent virus infections such as VZV leading to shingles [1, 38, 39]. We have previously observed that older adults have reduced recall responses to antigens such as VZV or candida, resulting in a reduced recruitment of T cells and dendritic cells at the site of antigen challenge [8]. This defect in immunity is not due to alterations in circulating antigen-specific cells but is a consequence of inflammatory defects in the skin environment [9, 36]. In this study, we confirm that there is a decreased recall responses in the skin of older adults as compared to young. The defect observed in the skin of older adults may be applicable to other tissue sites such as the lung and warrants further investigation.

Vitamin D insufficiency is increased in the older adult population [20] and is considered to be due in part to decreased outdoor activity and aging-related alterations in vitamin D metabolism [40]. In addition, vitamin D insufficiency in older adults is associated with frailty and increased systemic inflammation [19, 21]. Previous studies have shown that vitamin D_3_ supplementation in older adults with chronic inflammatory diseases such as osteoarthritis and heart failure significantly decreases the levels inflammatory mediators such as TNFα in the circulation [41, 42]. In contrast to these earlier studies, we did not observe significant decreases in circulating inflammatory mediators after vitamin D_3_ supplementation in the healthy volunteers with no overt inflammatory disease, consistent with data in an independent study of healthy older adults [43]. We did, however, observe that vitamin D_3_ supplementation was associated with a significant decrease in the non-specific inflammatory response to needle challenge in the skin.

Vitamin D has a plethora of effects on the immune system. Indeed, it is known that vitamin D enhances the number and function of Foxp3+ and IL-10+ Tregs [13–15], and thus Tregs could directly reduce non-specific inflammation observed in the skin after needle challenge. Another important function of vitamin D is that it enhances T cell receptor (TCR) signalling, as it increases expression of PLCy and facilitates activation of T cells in response to antigen [44], suggesting an additional mechanism by which vitamin D_3_ supplementation could be mediating the effects described in this study.

There were limitations to this study including the study size, gender distribution, and ethnic origin of the donors. Although this study had a higher proportion of female donors, we have previously observed that there is no significant difference in non-specific inflammatory response between males and females [12]. Our initial investigations in young and old individuals were carried out on people of diverse backgrounds and found no obvious difference between different racial groups [8, 9, 36]. However, this study was designed to be only carried out on Caucasians to exclude any potential effects of ethnic backgrounds. Further studies should now be performed to determine the impact of ethnicity, using our data on Caucasians as a reference point. As our study was an experimental study to establish mechanisms, rather than confirm the efficacy of vitamin D_3_, it will be important to do a larger study to assess the impact of vitamin D_3_ replacement on cutaneous immunity.

Another important health challenge within older populations is the reduction in vaccine efficacy with increasing age [3]. It has been proposed that inflammation has a detrimental effect on the functioning immune system and vaccine responses [7]. Therefore, there is a drive to develop therapies which can block inflammation to enhance vaccine responses. One such therapy that has been shown to improve influenza vaccine efficacy in older adults is the use of a TORC1 inhibitor. Inhibition of the mTOR pathway significantly enhances the immune response to vaccination and by doing so reduces influenza infections [45, 46]. We have also demonstrated that cutaneous immunity can be enhanced by a 4-day course of oral treatment with p38-MAPKinase inhibitor Losmapimod [8, 12]. However, the use of either inhibitor could potentially result in undesirable side effects, especially when used in the long term. In contrast, the use of vitamin D supplementation is safe, cheap, and readily available. Our data suggest that if used as part of a public health initiative targeting older adults, this has the potential to significantly improve the health span by improving antigen-specific immunity and increasing vaccine efficacy.

Vitamin D insufficiency has also been linked with worse clinical outcomes in the current COVID-19 pandemic [47]. Older people are more at a risk of increased morbidity and mortality from infection with the Sars-CoV-2 coronavirus [48]. Vitamin D is known to be important for respiratory health through the increasing production of antimicrobial peptides (such as cathelicidin) and reducing inflammation [17, 18, 49]. Therefore, vitamin D_3_ supplementation could be considered as a straightforward, cheap, and safe means to help improve immunity to SARs-CoV-2 infection.

Collectively, our data show that vitamin D_3_ supplementation could be a simple, cheap, and readily available therapy that could enhance antigen-specific immunity in older adults.

## Supplementary Material

ltaa008_suppl_Supplementary_Figures

ltaa008_suppl_Supplementary_Table_1

ltaa008_suppl_Supplementary_Table_2

ltaa008_suppl_Supplementary_Table_3

## Data Availability

RNAseq data relating to the young versus old comparison (Fig. 1) that support the findings of this data have been deposited on ArrayExpress accession number E-MTAB-9789. RNAseq data relating to the vitamin D_3_ replacement study that support the findings of this study have been deposited in NCBI Gene Expression Omnibus, https://www.ncbi.nlm.nih.gov/geo/query/acc.cgi?acc=GSE156212.

## Abbreviations

CRP: C reactive protein
25(OH)D: 25-hydroxyvitamin D
p38-MAPK: p38 mitogen-activated protein kinase
TPM: Transcripts per million
Tregs: T regulatory cells
T_RM_: Resident-memory T cells
VZV: varicella zoster virus
