# Supplementary material for: Vitamin D3 replacement enhances antigen-specific immunity in older adults
Source: Immunother Adv. 2020 Nov 25;1(1):ltaa008. doi: 10.1093/immadv/ltaa008 (PMC9585673; doi:10.1093/immadv/ltaa008)

**Supplementary Figure 1: pathways increased in VZV or Saline injected skin.**

Pathways upregulated in **A,** VZV-injected young skin 72 hours post-injection or **B,** Saline-injected old skin 6 hours post-injection. Ca2+ = Calcium; GM-CSF = granulocyte-macrophage colony-stimulating factor; GPCR = G-protein coupled receptor; HIV = human immunodeficiency viruses; IFN = interferon; IRF = Interferon regulatory factor; MDA = melanoma differentiation-associated protein; RIG-I = retinoic acid-inducible gene I; SCF-KIT = Stem cell factor-KIT; TRAF = TNF receptor-associated; VZV = varicella zoster virus; 6h = 6 hours.


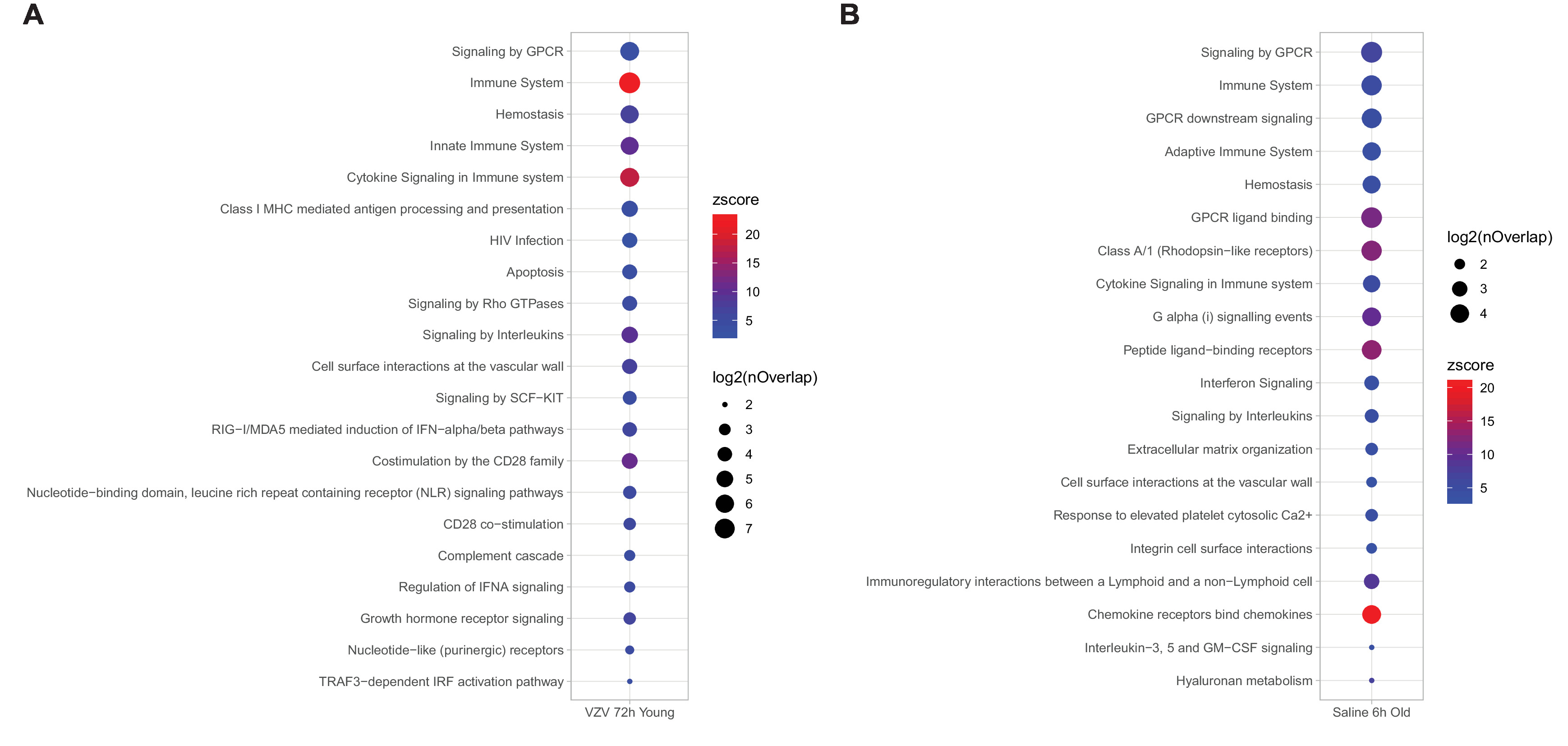


**Supplementary Figure 2: T cell-specific gene modules significantly correlates with VZV clinical score.**

Correlation between T cell-specific gene module and VZV clinical score. Analysed by Pearsons correlation test.


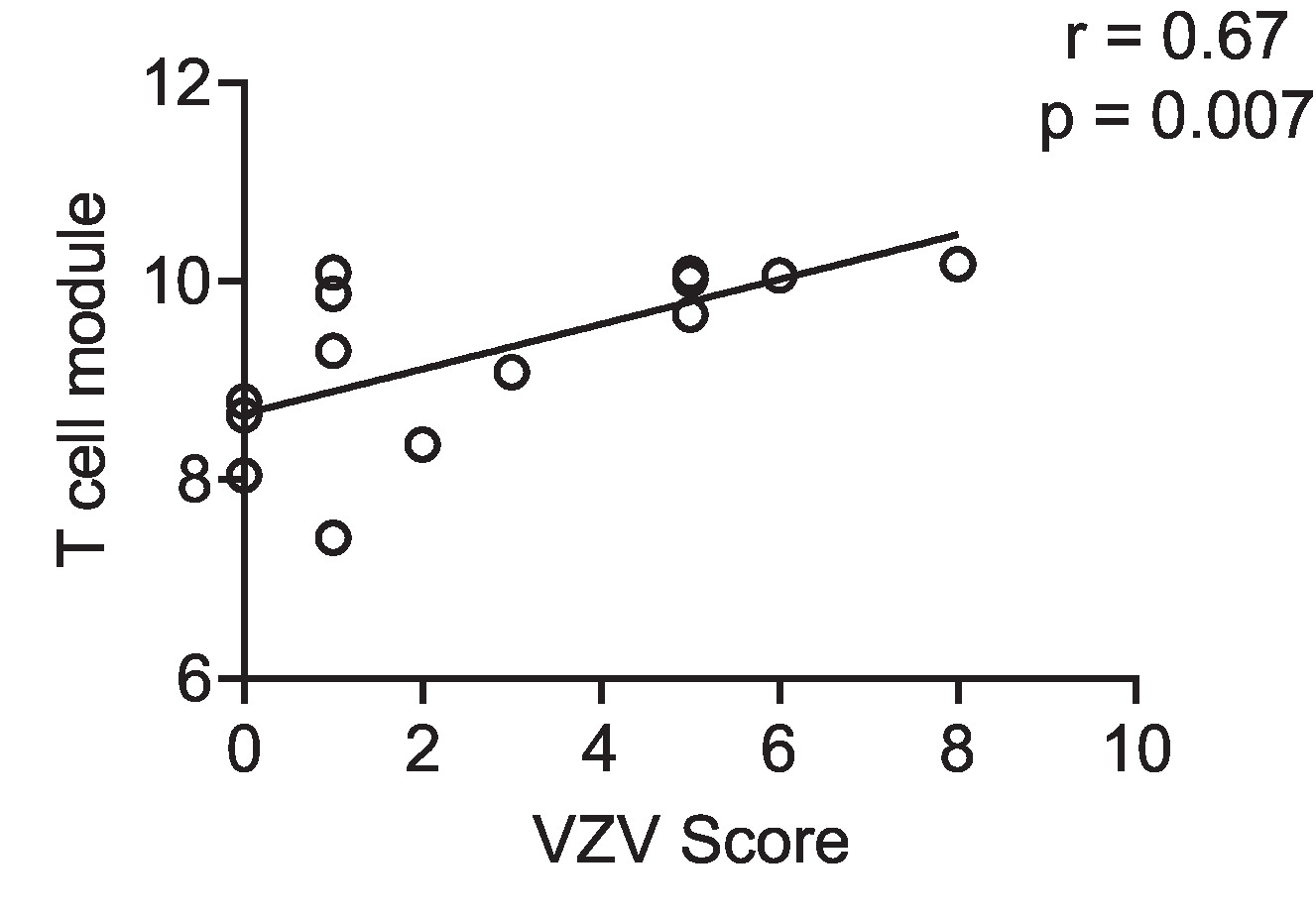

Supplement: ltaa008_suppl_Supplementary_Figures [file ltaa008_suppl_supplementary_figures.docx]
