# Supplementary material for: Vitamin D3 replacement enhances antigen-specific immunity in older adults
Source: Immunother Adv. 2020 Nov 25;1(1):ltaa008. doi: 10.1093/immadv/ltaa008 (PMC9585673; doi:10.1093/immadv/ltaa008)
Supplement: ltaa008_suppl_Supplementary_Table_1 [file ltaa008_suppl_supplementary_table_1.docx]

| **Inclusion Criteria** | **Exclusion Criteria** |
| --- | --- |
| Age >65 years. | Shingles vaccine, negative VZV serology |
| Healthy as determined by the investigator or medically qualified designee based on a medical evaluation including medical history, physical examination and laboratory tests. | HIV seropositive, known diabetes mellitus, current malignancy or any other history of neoplastic disease other than basal cell carcinoma in remission less than 1 year before recruitment, autoimmune diseases, keloid scarring, any skin disease |
| Subject is capable of giving written informed consent, which includes compliance with the requirements and restrictions listed in the ICF and is willing and able to return for all study visits. | Currently receiving any immunosuppressive therapy including corticosteroids, azathioprine, methotrexate tacrolimus or sirolimus, mycophenalate mofetil, Interferons, NSAIDS (excluding low dose aspirin), any antibody therapies or other biologics |
| Serum 25-hydroxyvitamin D <75 nmol/L | Any vaccination in 6 weeks before recruitment or plans for receiving a live attenuated vaccine during the vitamin D_3_ treatment period. |
| Serum corrected calcium concentration <2.65 mmol/L | Currently receiving any anticoagulant therapy |
| Estimated Glomerular Filtration rate (eGFR) >30 ml/min/1.73 m2 | Currently receiving phenytoin, barbiturate, cardiac glycoside, oral glucocorticoid or vitamin D_3_ supplement |
| Platelet count ≥150,000 per microliter | Other contra-indication to vitamin D_3_ supplementation: known sarcoidosis, known hyperparathyroidism or known nephrolithiasis |
| International Normalised Ratio (INR) <1.1 | Known allergy to vitamin D_3_ or its excipients |
| Activated Partial Thromboplastin Time (aPTT) ≤38 seconds | Use of another investigational product within 30 days or 5 half-lives (whichever is longer) or according to local regulations, or currently participating in a study of an investigational device. |
| **Supplementary Table 1 : Inclusion and exclusion criteria for the vitamin D_3_ clinical study** | History of anaphylactic reactions to local anaesthetics. |
